# Uncovering Protein Networks in Cardiovascular Proteomics

**DOI:** 10.1016/j.mcpro.2023.100607

**Published:** 2023-06-24

**Authors:** Maria Hasman, Manuel Mayr, Konstantinos Theofilatos

**Affiliations:** King’s British Heart Foundation Centre, Kings College London, London, United Kingdom

**Keywords:** protein regulatory networks, protein network reconstruction, cardiovascular proteomics, multi-omics, PTM-specific networks, network analysis, cardiac tissue protein networks, vascular tissue protein networks, matrisome protein networks

## Abstract

Biological networks have been widely used in many different diseases to identify potential biomarkers and design drug targets. In the present review, we describe the main computational techniques for reconstructing and analyzing different types of protein networks and summarize the previous applications of such techniques in cardiovascular diseases. Existing tools are critically compared, discussing when each method is preferred such as the use of co-expression networks for functional annotation of protein clusters and the use of directed networks for inferring regulatory associations. Finally, we are presenting examples of reconstructing protein networks of different types (regulatory, co-expression, and protein-protein interaction networks). We demonstrate the necessity to reconstruct networks separately for each cardiovascular tissue type and disease entity and provide illustrative examples of the importance of taking into consideration relevant post-translational modifications. Finally, we demonstrate and discuss how the findings of protein networks could be interpreted using single-cell RNA-sequencing data.

## Highlights

• Co-expression and PPI networks are widely used in cardiovascular research.
• Directed regulatory networks are more suitable for generating causality hypotheses.
• Tissue, cell, and disease status triggers rewiring of protein networks.
• Considering post-translational modifications can refine the reconstructed networks.
• Integrating protein networks with other omics data enables scientific deductions.

## Graphical Abstract

**Figure:**
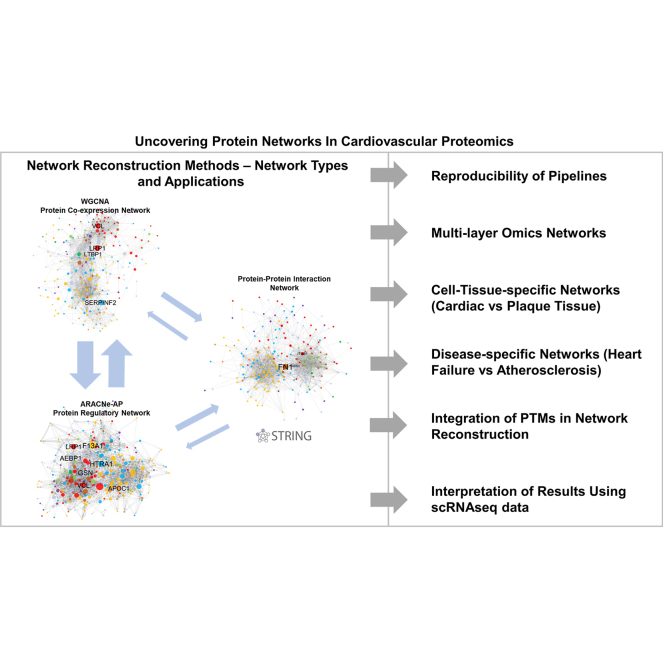

## In Brief

In the present article, we review and critically assess existing methods for reconstructing protein networks and discuss when each method is preferred for applications in cardiovascular research. We demonstrate the necessity to reconstruct networks separately for each cardiovascular tissue type and disease entity and provide illustrative examples of the importance of taking into consideration relevant post-translational modifications. Finally, we demonstrate and discuss how the findings of protein networks could be interpreted using single-cell RNA-sequencing data.

Biological networks have been widely used in many different diseases to identify potential biomarkers, causal genes, and drug targets (1). Genetic interaction networks (2, 3) have been widely used to map genetic mutations with phenotypic changes, but the focus of the present article is on protein network reconstruction from quantitative data. The basic types of protein networks are the experimentally or *in silico* reconstructed protein–protein interaction (PPI) networks and the functional networks, which could show similar protein co-regulation, expression, or function. The latter can be split into regulatory and co-expression networks, according to whether their edges are directed or not. Networks can reveal useful biological and molecular information by inspecting two different and complementary types of network properties, the topological and the functional ones (4). Topological characteristics are used to represent the structural features of the network and are associated with biological properties and certain parameters, such as the betweenness centrality, which is used to reveal critical nodes. The functional approach clusters the nodes based on their functional information, such as cell compartments and molecular functions (5). Recently, a systems-level approach, which involves focusing on a group of genes or proteins rather than on individual molecules, is being used to find mechanisms of complex diseases, which involve groups of genes or proteins (6). Several reviews are describing the different types of biological networks and analysis. Hu *et al.* (7) outlined different computational methods for identifying PPI networks. Vella *et al.* (8) described PPI and co-expression network reconstruction and analysis methods as well as studies involving the use of proteomics co-expression networks. Liu *et al.* (9) described different ways to identify critical nodes, and Meng *et al.* (10) described the different topological properties of PPI networks. Furthermore, previous reviews (11, 12, 13, 14, 15) have described the application of network analysis in diseases as well as systems biology approaches which include proteomics integration with other -omics technologies, network analysis, and their application in cardiovascular diseases (16, 17). To the best of our knowledge, however, none of them has focused on the different network types, the technical aspects of the network reconstruction methods, nor have they studied the effect of post-translational modifications and cell and tissue composition in the reconstructed networks. In this review, we discuss the role and types of protein networks, the different network analysis techniques, and tools, and focus on their application to tissue proteomics of clinical samples.

## Existing Methods for Protein Networks Reconstruction and Analysis

The basic steps of network analysis involve the reconstruction of PPI networks of undirected protein co-expression networks, and of directed protein regulatory networks, their clustering to identify significant modules, and their analysis and visualization to reveal key hub proteins that can serve as diagnostic, prognostic, or therapeutic biomarkers. PPI networks are static networks since they do not change, have the same connections between the nodes in all conditions, do not take into account the data of each experiment or different tissue or disease definition, and form a static integrated picture of protein activity. Table 1 presents the basic categories for protein network reconstruction and analysis, some indicative tools, and their advantages and disadvantages. PPIs can be divided into physical (direct) interactions and indirect interactions and both types of networks can be either reconstructed with experimental methods or predicted using *in silico* machine learning, mathematical modeling, or other methods (4, 12). There are many databases, where experimentally verified PPI networks can be retrieved, such as iRefIndex (18), combining information from primary databases, such as IntAct (19) and BioGRID (20). PPIs can also be computationally predicted (STRING (21) and Metascape (22)). Such databases have been extensively used for the reconstruction of PPI networks in several diseases (10, 23).

**Table 1:** Existing methods for the reconstruction and analysis of proteomic networks, indicative tools, and their implementations

Analysis type	Subcategory	Indicative methods	Advantages	Disadvantages	Implementation	Input data
Protein-Protein Interaction Network Reconstruction	Computationally and experimentally verified	STRING (21), Metascape (22)	i) Interactions for many species ii) Interactions from many sources iii) User-friendly network visualization and analysis	High false positive rate Not full coverage of interactomes	Webtool/Standalone (R, Bioconductor Package)	Single or multiple gene list
Protein co-expression networks	Correlation-based	WGCNA (24)	i) Sparse Networks ii) Optimized Threshold	i) One threshold for all nodes ii) Linear Associations Only iii) Undirected Networks	Standalone (R/Python)	i) Gene expression dataset ii) External trait data (eg clinical) to relate modules with
	Mutual Information Based	MIDER (25)	Distinguishes between direct and indirect links	Undirected Networks	Standalone (Matlab)	Time-series data
	Probabilistic	SEC (28)	Creates sparse matrices	The threshold needs to be decided with trial and error Undirected Networks	Standalone (Matlab)	Gene expression data
Protein Regulatory Networks	Mutual Information Based	ARACNe-AP (29)	i) Sparse Networks ii) Directed Networks iii) Non-linear associations iv) Removes indirect links	i) Cannot discriminate between ii) positive and negative associations iii) Requires a given set of transcription factors	Standalone (JAVA)	Gene expression data and tf/gene list
	Probabilistic	BNW (27)	i) Handles noise and uncertainty ii) Directed Networks	i) Does not support large networks ii) Feedback loops are not allowed iii) Static networks of ≤19 variables	Webtool	i) Hybrid datasets contain both continuous (eg gene expression data) and discrete (eg genotypes) variables ii) Can include prior knowledge
	Machine-learning based	dynGENIE3 (33)	i) Directed Networks ii) Good scalability	Semi-parametric	Standalone (Python/Matlab/R)	i) Steady -state and time series expression data ii) Can include prior knowledge (eg known TFs)
Network Clustering	Hard-clustering	HipMCL (88)	i) Fast Clustering in Large Networks ii) Supports edge-weighted graphs clustering	i) Does not allow unclustered nodes ii) Does not allow overlapping clusters	Standalone (C++)	Not Applicable
	Soft-clustering	ClusterOne (39)	i) Allows overlapping clusters ii) Allows for unclustered nodes iii) Does not allow small clusters	Lower Interpretability	Standalone (JAVA)/Cytoscape plug-in	Not Applicable

STRING is included in the computational methods of PPI network reconstruction as it uses an in-silico probabilistic method for PPI predictions.

The existing methods for co-expression network reconstruction were grouped into four basic categories: correlation-based, information theory-based, mathematical modeling, and other methods. Weighted gene correlation network analysis (WGCNA) (24) is one of the most widely used correlation-based approaches to construct co-expression networks. WGCNA provides tools to construct networks, identify modules, determine topological attributes, simulate, and visualize data. Correlation-based methods cannot model nonlinear associations. Thus, mutual information-based techniques, such as MIDER (25), have been introduced to address this issue. The problem of reconstructing co-expression networks is a mathematical optimization problem and this is the reason why mathematical and probabilistic modeling techniques, such as the Bayesian ones (26, 27) and the Sparse Estimation of the Correlation matrix (SEC) (28) have been widely used. However, these techniques suffer from a high number of assumptions and are not applicable to large-scale networks.

Protein regulatory networks are networks incorporating directionality and interaction type for each interaction. The existing methods for regulatory network reconstruction were grouped into three basic categories: information theory-based, mathematical modeling, and machine learning–based methods. One of the most widespread methods from this category is the Algorithm for the Reconstruction of Accurate Cellular Networks using adaptive partitioning strategy (ARACNe-AP) (29), which uses an information theoretic context based on the Data Processing Inequality (DPI) theorem to infer direct regulatory relations among transcriptional regulator proteins and target genes. ARACNe-AP estimates the mutual information threshold, uses bootstraps to reconstruct networks, and finally constructs a consensus network. Artificial Neural Networks (ANNs) can recognize any input pattern entered and create models of the data structure and connections that occurred during the procedure. Recurrent neural networks are widely presented as the most effective neural network-based models for gene regulatory network construction because of their ability to represent and model feedback and memory mechanisms (30). Rubiolo *et al.* (31) have used the Extreme Learning Machine (ELM)–supervised neural model to reconstruct regulatory networks from time series. Machine learning models have also been suggested to reconstruct protein regulatory networks. Such an example is GEne Network Inference with Ensemble of trees (GENIE3) (32), an unsupervised method for network inference based on regression trees. This method is scalable, suitable for non-linear data, and efficient in the case of many features. However, it only takes steady-state data as input. Thus, Huynh-Tu and Geurts (33) proposed an adaptation, the dynamic GENIE3 (dynGENIE3), which can combine both steady-state and time series data as an input. Keyl *et al.* (34) used a hybrid method that combines neural networks and a machine learning technique, layer-wise relevance propagation (LRP), to predict patient-level proteomic networks. Their method first uses neural networks to predict protein abundances and after training the network, it uses LRP to identify the input variables of the neural network that contributed the most to the prediction of the output.

Clustering is the first step after constructing and visualizing the network (with the most popular and widely used tool for visualization being Cytoscape (35)). Network clustering is used to group proteins with similar expression patterns and results into groups of nodes that often correspond to different functional groups. Clustering methods can be separated into three categories: (1) Hard clustering techniques, such as Markov Clustering (MCL) (36), and Restricted Neighborhood Search Clustering Algorithm (RNSC) (37), where all proteins are clustered and each protein can only belong to one cluster; (2) Soft clustering techniques, such as Molecular Complex Detection (MCODE) (38), ClusterONE (39) and WRNSC (40), where clusters can overlap and some proteins could remain unclustered; (3) Clustering techniques that are based on modularity density metric to detect communities, such as Louvain Clustering (41, 42). The Louvain clustering algorithm has found widespread use in various forms and has been applied to numerous biological networks. However, modularity-based algorithms like Louvain have a limitation in that they tend to exclude communities below a certain size threshold, known as the resolution limit. To overcome this limitation, a recent publication (43) used a recursive hierarchical approach in combination with the Louvain community detection algorithm to enable the resolution of hierarchically nested structures. Hierarchical clustering methods belong to the hard clustering techniques but are also able to provide a hierarchical organization among the proteins of a protein network (44). When all these network clustering methods are applied to physical PPI networks, the predicted clusters correspond to protein complexes (7). However, recently, these techniques for protein complex prediction were complemented with methods taking into consideration computationally predicted protein structure (45) or being applied directly to raw mass spectrometry data (46). Furthermore, another group of methods has been introduced to hierarchically organize networks (47) and to hierarchically organize tissues based on their underlying proteomic networks (48).

Another important analysis step involves the identification of essential nodes, also called hubs. Hub nodes are central nodes to the network and could be separated into intra- and intermodular hubs. Intra-modular hubs are central to each network cluster they belong to, whereas inter-modular hubs are central to the whole network (13). The most widely used methods for identifying hubs in a network are based on topological centralities such as the betweenness, degree, PageRank, and clustering coefficient with many tools, including Cytoscape (35), Network Analysis Tools (NeAT) (49) and NetConfer (50) enabling such analysis.

## Network Approaches for Tissue and Disease-Specific Networks

The PPI databases can provide only a static form of the proteome interactome and not the cell- and tissue-specific context-dependent proteome connectivity (51). Cell-specific interaction networks can reveal characteristic biological properties and unique interactions of each cell, which are often related to distinct phenotypes (52). It has been observed among different cell-specific networks that, even when their interactomes share most of the proteins, the interactions among them differ. These interactome differences reveal cell-specific processes and are a result of distinct mechanisms, such as differential protein abundance, protein localization, or post-translational modifications (52, 53, 54). This cellular diversity leads to tissue-scale protein interactions. Attempts to report tissue-specific protein interactomes include TissueNet (55) a web tool that contains tissue-specific PPIs for human proteins and displays the expression of a query protein and all its interaction partners in the different tissues available. In the context of disease, Basha *et al.* (56) used MyProteinNet (57) to show tissue selectivity for gene expression and interactomes, exploring various tissues and including heart tissue. Greene *et al.* (53) used a kidney-specific network reconstructed with the NetWAS tool to train a classifier that can identify tissue-specific connectivity patterns associated with hypertension.

**Fig. 1:**
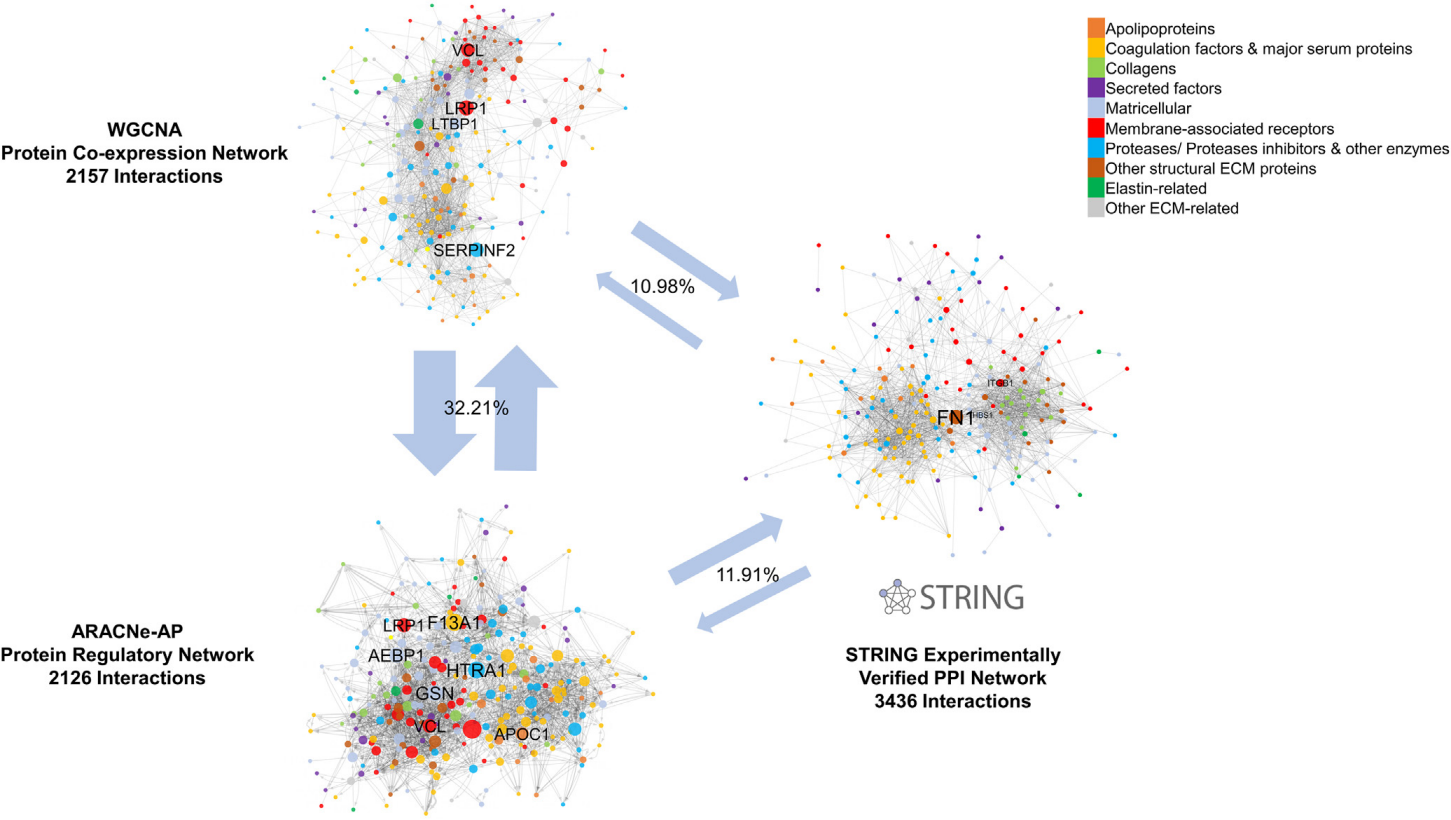
**Different networks to capture different aspects of the matrisome network of atherosclerotic plaques.** Example of different types of networks for the matrisome of human *left* anterior descending coronary artery. Data-independent acquisition mass spectrometry data of 99 samples were used from the Parker *et al.* study (80) and filtered to keep only matrisome-related proteins, according to a custom matrisome database composed of extracellular proteins from the MatrisomeDB (81), apolipoproteins, and other secreted proteins that are consistently quantified (less than 20% missing values). The experimentally verified protein-protein interaction network was created by mining matrisome interactions from the STRING web tool (21). The protein co-expression network was reconstructed using the WGCNA pipeline (24), with Pearson’s correlation as the interaction metric and 0.5 with a soft power of 10 as a threshold to infer interactions. ARACNe-AP (29) with default parameters was used to reconstruct the protein regulatory network with the same data. All networks were visualized using Cytoscape (35), proteins were colored based on the matrisome group they belong to, and node size was set to be proportional to the betweenness centrality of the node in the network. Only the nodes with betweenness centrality above 0.05 were labeled. The confirmed interactions of a network against another type of network are depicted with *arrows* connecting the different networks, and the average percentages (%) of common interactions between the two types of networks are also depicted. AEBP1, Adipocyte enhancer-binding protein one; APOC1, Apolipoprotein C-I; F13A1, Coagulation factor XIII A chain; FN1, Fibronectin; GSN, Gelsolin; HTRA1, Serine protease HTRA1; ITGB1, Integrin beta-1; LRP1, Prolow-density lipoprotein receptor-related protein one; LTBP1, Latent-transforming growth factor beta-binding protein one; SERPINF2: Alpha-2-antiplasmin; THBS1, Thrombospondin-1; VCL, Vinculin.

Figure 1 demonstrates a visual example of reconstructing the basic types of networks presented in Table 1 (PPI, protein co-expression, and protein regulatory networks). We have chosen a proteomics dataset for the matrisome of the left anterior descending coronary human arteries, using the most widely used method from each category. The matrisome was defined as the ensemble of Extracellular Matrix (ECM) and ECM-associated proteins. The ECM is a three-dimensional structure present in all tissues but different for each organ. It is composed of proteins such as proteoglycans, collagens, and glycoproteins (58). ECM remodeling in atherosclerosis plays an important role in plaque destabilization and progression (59). Bayesian methods and other mathematical modeling methods were not used since the examined tools (Table 1) were not supporting datasets of this sample and protein markers size. Limited overlap was found between the static PPI network and the reconstructed networks, as the percentages of confirmed interactions of the PPI network against both the protein co-expression (8.47%) and the protein regulatory (9.11%) network was less than 10%. PPI networks, even the ones based on experimental evidence, are being created based on evidence from different types of tissues and conditions, and it is highly likely that most of them are not relevant to a particular tissue. As shown in Figure 1, significant hub proteins (betweenness centrality over 0.05) in the PPI network, such as Fibronectin 1 (FN1), were not returned as significant in the other networks. Protein co-expression and protein regulatory networks presented higher overlap, with more than 30% of the interactions of one network confirmed in the other (31.99% and 32.42% respectively). Moreover, significant overlap was observed in the hub proteins, with the membrane-associated proteins Vinculin (*VCL*) and *LRP1* (LDL Receptor Related Protein 1) being hubs (betweenness centrality over 0.05) for both protein co-expression and regulatory networks. Further experimental validation should be performed to verify interactions and significant proteins experimentally by performing pull-down assay experiments with the revealed hub proteins and their candidate interaction partners. Moreover, instead of just exploring the static PPI networks, this analysis should be complemented with reconstruction and analysis of co-expression and regulatory networks in the specific tissues of interest to probe real interactions and disease mechanisms.

**Fig. 2:**
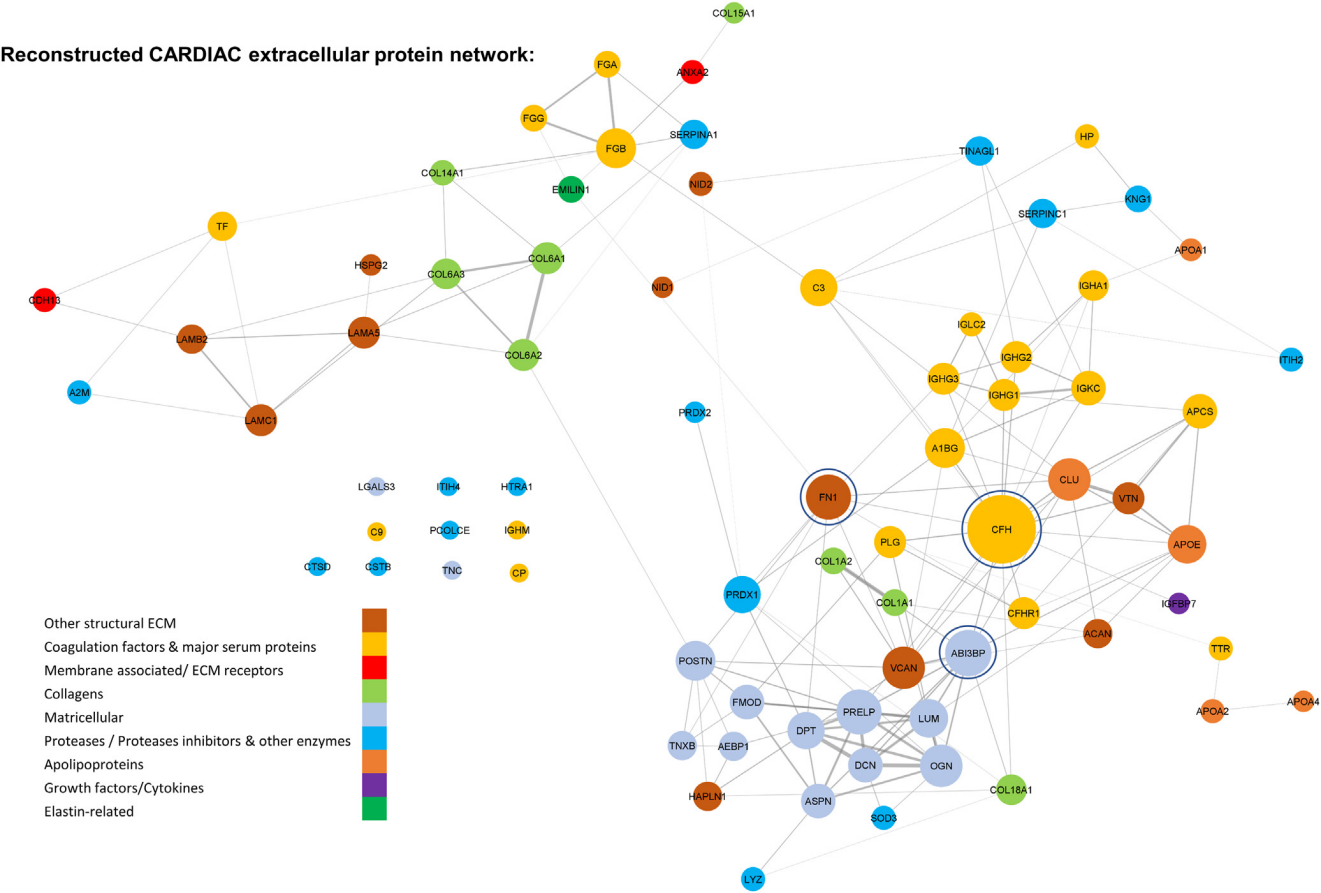
**Heart tissue matrisome network.** Label-free discovery mass spectrometry data of 65 ischemic heart tissue samples were used from the study by Barallobre-Barreiro *et al.* (60) and filtered to keep only matrisome-related proteins, according to a custom matrisome database composed of extracellular proteins from the MatrisomeDB (81), apolipoproteins, and other secreted proteins that are consistently quantified (less than 30% missing values). ARACNe-AP (29) with default parameters was used to reconstruct the regulatory network, filtering out negative associations using the SIREN algorithm (82). Networks were visualized using Cytoscape (35), matrisome proteins were *colored* based on the functional category they belong to, edge width was set to be proportional to mutual information metric, node size was set to be proportional to its degree centrality and hub proteins are highlighted in *blue*.

**Fig. 3:**
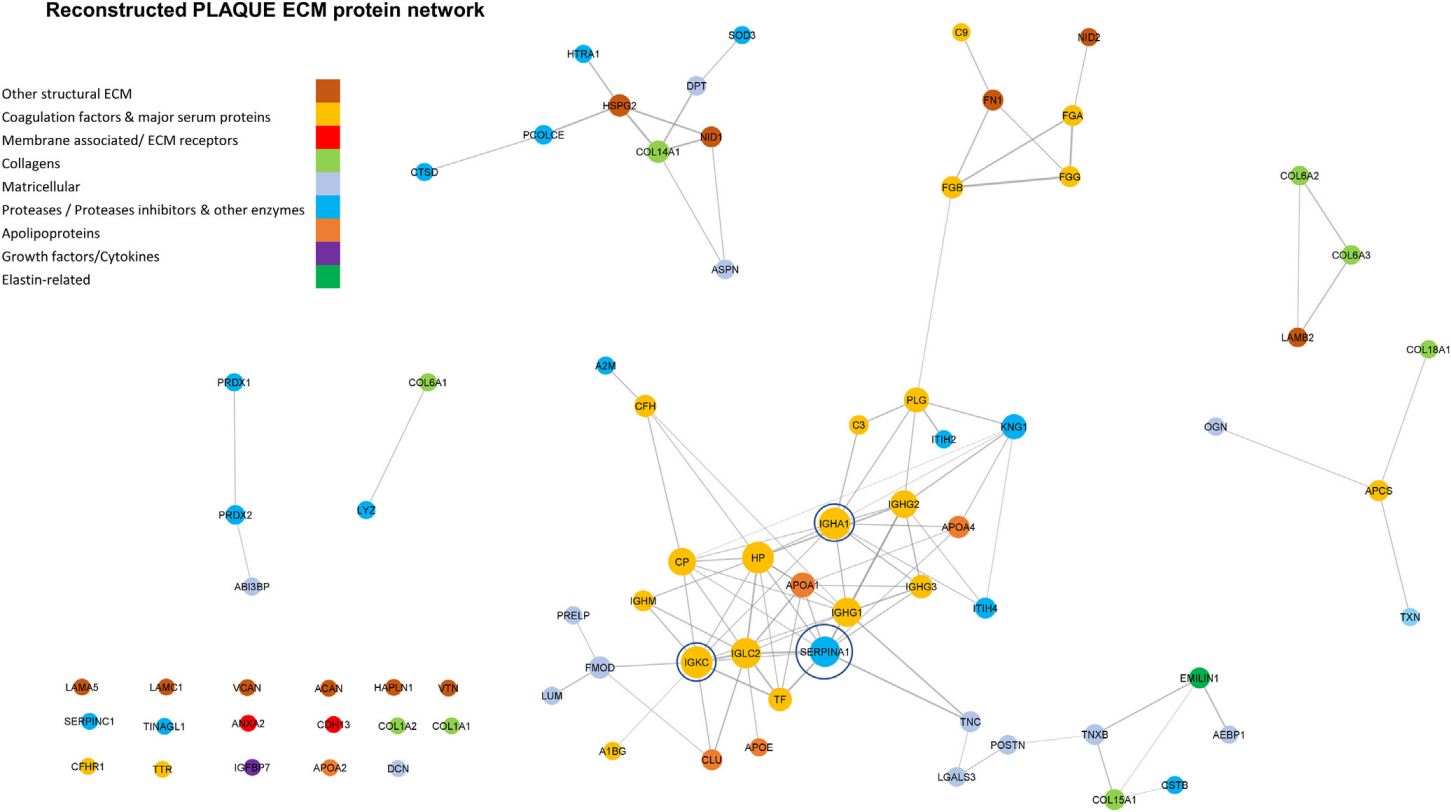
**Carotid plaque matrisome network.** Label-free discovery mass spectrometry data of 12 carotid endarterectomy samples were used from the study by Langley *et al.* (61) and filtered to keep only matrisome-related proteins, according to a custom matrisome database composed of extracellular proteins from the MatrisomeDB (81), apolipoproteins, and other secreted proteins that are consistently quantified (less than 30% missing values). ARACNe-AP (29) with default parameters was used to reconstruct the regulatory network, filtering out negative associations using the SIREN algorithm (82). Networks were visualized using Cytoscape (35), matrisome proteins were *colored* based on the functional category they belong to, edge width was set to be proportional to mutual information metric and node size was set to be proportional to its degree centrality.

To illustrate the tissue and disease specificity of the reconstructed networks, we reconstructed two networks of the extracellular matrisome using label-free proteomics data from samples of previously published studies on ischemic heart failure (60) (Fig. 2) and atherosclerotic carotid plaques (61) (Fig. 3). For the reconstruction of these networks, we used only extracellular proteins that were consistently quantified in both datasets (<30% missing values per protein). Both networks were reconstructed using the ARACNe-AP information theory-based method with default parameters for network reconstruction (100 bootstraps for creating the network and Bonferroni correction of the nominal *p*-values of each inferred edge). With the network reconstruction, we were able to confirm known interactions in the heart tissue network such as the ones between collagens (*COL6A1, COL6A2, COL6A3*) and the ones between laminins (*LAMA5, LAMB2, LAMC1*). We performed network analysis for the two networks, using the NetworkAnalyzer (62) Cytoscape plug-in. The power law distribution was fitted to both networks’ degree distribution, verifying the scale-free topology of the networks. The networks for plaque and heart tissue had comparable clustering coefficients of 0.24 and 0.27 respectively, suggesting a similar topology for both networks. However, the two disease- and tissue-specific networks differed in several other properties. For example, the heart tissue network has higher connectivity and a smaller number of sub-networks than the carotid plaque network, with 11 connected components and a radius of 5, compared to 23 connected components and a radius of 1. These differences suggest that the two networks have distinct structures and may be governed by different underlying biological processes, in particular, higher connectivity among the matricellular proteins in the ischemic heart tissue network compared to carotid plaques. In contrast, serum proteins and proteases are more central in the carotid plaque network since carotid endarterectomy lesions are expected to contain more serum-derived proteins than myocardial tissue. Clustering analysis (using the clusterONE Cytoscape plug-in, a minimum number of five proteins per cluster, and the MI) also verified this, with certain matricellular proteins forming a unique cluster for the cardiac tissue network that was not present in the carotid plaque network.

**Fig. 4:**
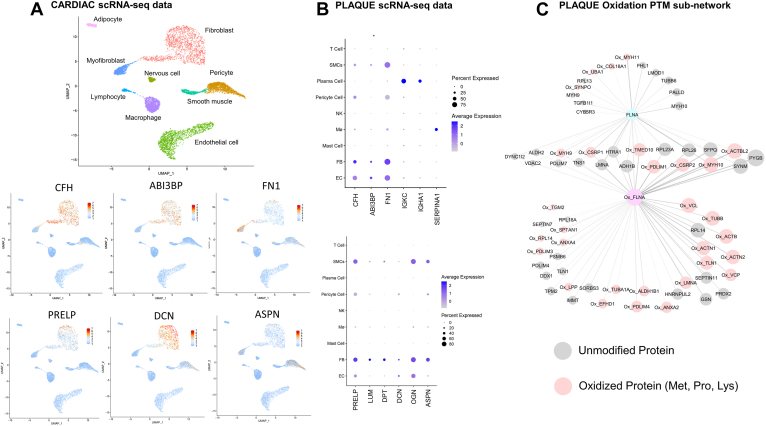
**Interpreting cardiac and plaque protein networks using scRNAseq data and the effect of oxidation PTM in the interactions of Filamin-A (*FLNA*).***A*, uniform manifold approximation (UMAP) feature plots of the expression of the *top* central nodes (hubs) of the network and other central proteoglycans, using the Hocker *et al.* single-cell RNA-seq dataset (83) of human heart tissue samples (8993 cells from two healthy donors) from the ExpressHeart web portal (84). *B*, expression *dot* plots of *top* three central proteins (hubs) in the carotid plaques network and the cardiac tissue network and other central in the cardiac protein network proteoglycans (Fig. 2) using the PlaqView web tool (85), scRNA-seq data from carotid plaques (Pan *et al.* dataset (86), n = 3) and Aran *et al.* method (87) to label cell clusters. *C*, label-free discovery mass spectrometry data of 12 carotid endarterectomy samples were used from the study of Langley *et al.* (61). Data was exported in peptide level from Proteome Discoverer software and oxidation of proline, lysine, and methionine was used as a dynamic modification. The network was reconstructed using the WGCNA method (24) with the Pearson’s correlation method, a first soft power of 10 and a final hard threshold of 0.25. Nodes are proportional to the degree of each protein (based on all proteins network), edge width is proportional to the MI metric, and node *colors* are set according to the oxidation modification (*light grey* for unmodified proteins and *pink* for oxidized proteins, with the unmodified and oxidized form of FLNA having *light blue* and *light purple* color respectively). EC, Endothelial cells; FB, Fibroblast cells; Mo, Monocytes/macrophages; NK, Natural Killer cells; SMCs, Smooth Muscle Cells.

The most significant difference is observed in the top hub proteins using the degree centrality of each network. Complement Factor H (*CHF*), Fibronectin (*FN1*), and Target of Nesh-SH3 (ABI3BP) are the top three interconnected proteins of the heart tissue network (with their degree centrality being 40, 23, and 22 respectively). Publicly available single-cell RNA-sequencing data from heart tissue samples (Fig. 4*A*, ExpressHeart web portal) showed that these matricellular and structural ECM proteins are mostly expressed in fibroblast and myofibroblast cells in the heart. It is noteworthy that cardiomyocytes were not included in this analysis, as the ExpressHeart web portal is only based on scRNAseq and does not include single nuclei data, thus cardiomyocytes are too big to detect with this technique. On the contrary, these proteins lost their central role in the carotid plaque network and stopped being hubs (Fig. 3), having a very smaller degree centrality, belonging to different subnetworks and are expressed in both smooth muscle and endothelial cells (Fig. 4*B*). Immunoglobulins (Immunoglobulin Kappa Constant: *IGKC*, Immunoglobulin Heavy Constant Alpha 1: *IGHA1*) and serpin family A member 1 (*SERPINA1*) were the top three hub proteins in plaques (with a degree of 11 for immunoglobulins and 10 for SERPINA1 respectively) and were highly expressed in plasma cells and monocytes/macrophages, respectively (Fig. 4*B*). Moreover, proteins with similar functionality were more closely connected in the heart tissue network than the carotid plaques one. One such example is the matricellular proteins and especially the proteoglycans (*DPT, PRELP, LUM, DCN, OGN, ASPN*), which form a highly connected network component in the heart tissue network (Fig. 2), whereas plaques showed less interconnectivity and belonged to different subnetworks. In opposite to their cellular expression in the heart tissue (myofibroblasts and fibroblasts), in plaques these proteins were not only expressed in fibroblasts but also in endothelial and smooth muscle cells (Fig. 4*B*), reflecting the higher cell heterogeneity of carotid plaques. Thus, the combination of network analysis and single-cell RNA-sequencing data verified that the known cell composition differences between the two tissues and diseases are reflected in the reconstructed networks and identified different hub proteins for each matrisome network.

## Integration of PTMs in Network Reconstruction

Post-translational modifications (PTMs) are playing an important role in the structure and function of proteins and should be taken into consideration when reconstructing biological networks using proteomics data. Some tools already take PTMs in network reconstruction into consideration, such as the iPTMnet (63).

To assess the influence of PTMs, we reconstructed a protein regulatory network for atherosclerotic plaques based on the same dataset from Figure 3 but considering oxidized and unoxidized forms of each protein as separate nodes. To explore the effect of oxidation on the interactions and correlation patterns of the proteins in human atherosclerotic plaques, we exported the atherosclerotic dataset at the peptide level, choosing the oxidation of methionine, proline, and lysine residues as a dynamic modification. We used the consistently detected peptides (<30% missing values) to quantify the unmodified (at least two peptides per protein) and oxidized proteins. The WGCNA method (24) was used for network reconstruction. Filamin A (*FLNA*) was among the proteins which presented the biggest differentiation in its degree centrality when compared with its oxidized and unmodified form (54 and 34-degree centrality, respectively). Filamin A is a cellular protein that crosslinks actin filaments and links them to membrane glycoproteins and is involved in the remodeling of the cytoskeleton, to effect changes in cell shape and cell migration. Filamin A is also secreted, and it was consistently quantified in the extracellular extracts of the carotid plaque dataset (64). Seven *FLNA* peptides were found to be oxidized, with four of them being oxidized in methionine and three of them in proline amino acids. Accurate quantification of methionine oxidation in a proteomics scale has technical limitations as methionine could be readily oxidized during sample preparation (65). However, the sum of the abundances of methionine oxidized peptides was highly correlated with the sum of proline oxidized peptides of *FLNA* (Spearman’s Rho: 0.97). Thus, we included methionine peptides. As shown in Figure 4*C*, the oxidized and non-oxidized forms of *FLNA* share common interactors, but each form has also unique interactors. The unique interactors of oxidized *FLNA* are enriched in cellular responses related to cell-cell communication, neutrophil degranulation, and smooth muscle cell contraction.

## Multilayer-Omics Networks

Exploring the interconnectivity of the different molecules that constitute cells, that is, proteins, genes, and metabolites, could enable capturing more complex cellular mechanisms in comparison to separately studying each omics modality. Hammoud and Kramer (66) have recently reviewed such networks and their application to PPI, cell, and gene expression networks. Such an example of a multi-omics network reconstruction software is COSMOS (67), which combines transcriptomics, phosphoproteomics, and metabolomics datasets to build a multi-omics network. Using prior knowledge, this method first builds networks from omics datasets from public databases, then refines those networks by removing the edges that create incorrect predictions when applied to only one omics modality and, finally, further filters the network based on only differentially expressed genes, proteins, and metabolites. The resulting network consists of the differentially expressed molecules and their interactors in a set number of regulatory steps away, creating a causal network.

## Reproducibility and Methods Sharing

As highlighted in Table 1, various methods are available for reconstructing different types of networks using proteomics data. These methods often require different programming languages, input and output file formats, and software package versions. In addition, different operating system requirements can further complicate the use of these tools and the reproducibility of results and analysis. To address this problem and ensure reproducibility, Netbooks (68) have been developed either based on Jupyter Notebooks (69) or other markdown formats, including R Markdown (70). Jupyter Notebook is a web-based, open-source application originally designed for Python programming, but it now supports over 50 programming languages. With Jupyter Notebook, users can create and share documents that contain text, code, and other project-related materials. These documents, known as notebooks, can include various types of output, such as plots, interactive graphics, and other visualizations, and can be easily manipulated using third-party software tools.

## Conclusion

The reconstruction and study of different types of disease networks, as demonstrated in this review article, is mostly based on the reconstruction of co-expression networks using established pipelines such as WGCNA, or on the use of static commercial (*e.g.* IPA) or publicly available (*e.g.* STRING) PPI networks. Despite their simplicity and ease of use, they have their limitations. Many researchers try to overcome this issue by combining proteomics with genomics data in an attempt to identify pQTL (71, 72), but these approaches are limited to genomic variants which alter protein abundances and, thus, fail to capture pathogenic mechanisms related to post-translational modifications, protein degradation, and protein-protein interactions. To overcome these limitations, more robust pipelines and methods are needed to reconstruct and study directed regulatory networks, which are more suitable for generating and validating causality hypotheses and performing simulations. Moreover, multi-omics networks combining RNA and protein networks (17, 73) and the integration of single-cell RNA-sequencing and proteomics data (74), further allow the parallel analysis of transcriptional and translational mechanisms, but this analysis requires the development of new methods, such as the consensus clustering (75) that has been recently introduced.

Another important aspect is the validation of these network findings. Network analysis is a strong tool for hypothesis generation but its application on direct or indirect interactions between proteins should be further validated with additional experimental techniques. Two-hybrid screening is a widely used method to detect binary interactions in eukaryotic cells. One of the main limitations of this technique is that it can generate false positives or false negatives. Other techniques for experimentally validating PPI networks at scale include MS-based techniques. In affinity purification (AP-MS) a bait protein is purified with antibodies along with its potential interactor partners and these purified proteins are then identified by MS. In cross-linking MS (XL-MS), a protein mixture is incubated with chemical cross-linkers and then analyzed by LC-MS/MS for cross-linked peptides. Further support can be derived from co-fractionation experiments coupled to MS, where samples are lysed, and fractionated, and each fraction is analyzed with LC-MS/MS and protein abundances are plotted across fractions. Co-fractionation, however, is no proof for genuine protein interactions but can be considered as supporting evidence for an interactome network (76). The development of cross-linking mass spectrometry technologies (77) has allowed for the validation of protein-protein interactions on large scale, while single-cell proteomics (78, 79) can provide an additional layer of validation for the findings inferred by combining protein networks and single-cell RNA-sequencing data.

The network reconstruction process is highly affected by covariates and medications should thus be taken into consideration when reconstructing networks and when interpreting findings based on them. Moreover, as protein interaction networks are significantly different among different diseases and sample types, with cell composition being one of the most important factors. Thus, it is paramount that networks should be reconstructed specifically for each tissue type and disease entity, while when proteomics data are used for reconstructing the networks post-translational modifications should be taken into consideration as illustrated in the example provided in the present manuscript for carotid plaque networks. With all these factors taken into consideration, the comparisons of these networks become more meaningful with regard to identifying potential pathophysiological mechanisms that can then be further validated experimentally.

## Conflict of interest

The authors declare that they have no known competing financial interests or personal relationships that could have appeared to influence the work reported in this paper
